# Effect of Keller Funnel on the Rate of Capsular Contracture in Periareolar Breast Augmentation

**DOI:** 10.1097/GOX.0000000000001834

**Published:** 2018-06-18

**Authors:** Ashley N. Newman, Steven P. Davison

**Affiliations:** From the Georgetown University School of Medicine, Georgetown University, Washington, D.C.

## Abstract

**Background:**

Capsular contracture is 1 of the most common complications after breast implant surgery and is a major indication for reoperation. Capsular contracture is believed to be a multifactorial process that is affected by implant texture, incision type, and ultimately pocket contamination. This contamination causes a biofilm that leads to capsular contracture. The intraoperative use of a Keller funnel is a mechanical way to decrease the implant’s contact with the skin and ducts, reducing bacterial contamination that can cause these biofilms. For this reason, periareolar breast augmentation has been less popular among surgeons. The purpose of this study was to examine if there was a significant difference between the rates of capsular contracture in patients having periareolar breast augmentations with the use of a Keller funnel for insertion and those having periareolar breast augmentations without Funnel use.

**Methods:**

This level 3 retrospective study followed 2 groups of patients, the first having periareolar breast augmentations without the use of a funnel for insertion (group A; patients n = 15; implants n = 30) and the second having periareolar breast augmentations with the use of a funnel for insertion (group B; patients n = 151; implants n = 300).

**Results:**

The rate of capsular contracture in group A was found to be 10% compared with a rate of capsular contracture of 1.3% for patients in group B, an 87% reduction (*P* = 0.0019).

**Conclusions:**

According to the results found in this study, the rate of capsular contracture in patients having periareolar breast augmentations after insertion with a Keller funnel was statistically significantly lower than the rate in patients having implants inserted without the assistance of a funnel, making the device useful in reducing the occurrence of postoperative capsular contracture.

## INTRODUCTION

**Figure 1.**
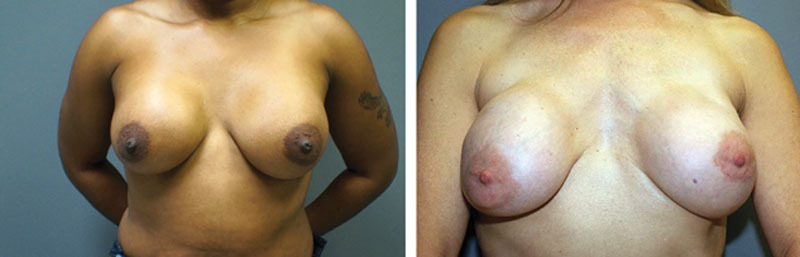
Grade III capsular contracture of the right breast 2 years postoperatively on set unknown (A); grade IV capsular contracture of the bilateral breasts 17 years postoperatively, onset unknown (B).

Capsular contracture is the leading complication after breast augmentation. The contracture is thought to be caused by a low-grade bacterial infection or the formation of a biofilm around implants that causes severe inflammation.^1,2^ The main bacterium found in capsules is *Staphylococcus epidermidis*, which is present on the skin and in the breast ductal secretions.^2–10^ Although bacterial contamination has been implicated in capsule formation, the process of contracture is said to be multifactorial, likely including inflammatory responses of the immune system.^1,4,11,12^ Inflammatory processes stimulate the release of proinflammatory cytokines that promote the accumulation of effector T-cells in capsules and, at the same time, inhibit peripheral regulatory T-cell generation. It is hypothesized that there is also a possible conversion of existing regulatory T-cells into effector T-cells by cytokines, which can upset the balance between protective and proinflammatory cells within a capsule.^4,13^ Although not proven, these more recent hypotheses aim to explain why with recent developments in no-touch techniques, capsular contracture can still occur. Researchers have also proposed that collagen deposition is a risk factor for the onset of capsular contracture.^14^ Most grade I and II capsular contractures are considered normal and can be addressed using massage techniques and Singulair anti-inflammatory medication, whereas grade III and IV contractures are considered to be abnormal and usually result in the need for surgical intervention (Fig. 1).^15,16^ Stutman et al.^17^ observe that the majority of capsular contractures in breast augmentations occur within the first 12 months (56% for periareolar excision).^18^

There are multiple factors that contribute to the onset of capsular contracture. Among these are implant texture, implant type, incision type, rupture/leakage, and pocket contamination with blood, bacteria, and synthetic fibers.^19,20^ Many of these factors can be controlled for by using antimicrobial implant baths and pocket irrigation, along with refining surgical techniques to minimize the implant’s contact with the surgeon’s gloves and patient’s skin. The use of triple-antibiotic breast pocket irrigation has been proven to greatly reduce the incidence of capsular contracture in breast augmentations.^21^ It has been found that periareolar breast augmentations have an increased incidence of capsular contracture compared with other incision types such as inframammary fold breast augmentations (9.5% compared with 0.59%, respectively, given implant placement with ideal surgical technique).^15,22,23,32^

The development of the Keller Funnel, a mechanical insertion device, in 2009 maximized a no-touch implant technique by giving an alternative to hand-placement of implants into breast pockets.^24^ The funnel is constructed of polymeric vinyl with a lubricous hydrophilic coating. The funnel is cut to implant size and then hydrated before the implant is poured directly from the packaging into the funnel. Finally, the funnel is placed about 1 cm inside the dissected pocket, and the implant is advanced through the funnel into the pocket as a no-skin touch technique.

The funnel makes implant insertion safer by decreasing the shell trauma to the implant, the contact with the patient’s skin, and the contact with the surgeon’s gloves during insertion. The funnel has been experimentally shown to reduce skin contact and contamination by 27-fold (*P* = 0.00059).^22,25^

This present study aims to explore the statistical significance between the rates of capsular contracture in patient’s having periareolar breast augmentations using the no-touch, Keller funnel technique, and those having periareolar breast augmentations, but with conventional no-touch precautions^29^.

**Null hypothesis:** There is no statistically significant difference between the rates of capsular contracture in patients having periareolar breast augmentation with the use of a Keller funnel and those without the use of a Keller funnel for insertion.

**Experimental Hypothesis**: The rate of capsular contracture in patients having periareolar breast augmentations with the use of a Keller funnel for insertion is statistically lower than patients having periareolar breast augmentations without the use of a Keller funnel for insertion.

## METHODS

The records for 237 patients having periareolar breast augmentations, from December 10, 2010, to August 31, 2017, with the assistance of a Keller funnel, were reviewed. Patients having silicone and saline implants placed were included and followed to determine the rate of capsular contracture in periareolar breast augmentations. Twenty patients having periareolar breast augmentations between July 1, 2008, and December 9, 2010, without a Keller funnel, but using conventional change in glove and tegaderm barrier no touch precautions, were followed to examine the baseline rate of capsular contracture for the practice. Before 2014, any augmentations done with the use of a Keller funnel were done with the KF-1 type Keller funnel (73 of the augmentations included in this study). Upon its release in 2014, augmentations were done with the KF-2 type Keller funnel (93 of the augmentations included in this study). All patients were skin prepped with betadine solution, and pockets were irrigated using a single antibiotic rinse (clindamycin with saline).

Each patient included in the study was contacted by phone or e-mail and asked if they had had any complications regarding their breast augmentation including hardening, unevenness, pain, general discomfort, reaugmentation, and so on. Clinical records, photographs, and postaugmentation visits were reviewed. Patients were asked to reply with images if possible and were also sent the article “*Capsular contracture: what is it? What causes it? How can it be prevented and managed?”.*^15^ Any patient reporting they were unsure about whether or not they had any type of contracture was asked to come into the office for clinical examination. Any patient who was unsure and could not come in to the office to be evaluated was excluded from the study (n = 21). Patients who had been in for a follow-up, 3 or more years postoperatively were not contacted. To be included in the study, patients had to be at least 1 year postoperative from the date of implantation.

Patients were placed in 1 of 2 groups for analysis. Group A consisted of patients having periareolar breast augmentations without the use of a Keller funnel for insertion. Group B consisted of patients who had periareolar breast augmentations in which a Keller funnel was used for insertion. To control for variations, the sole surgical practice affecting placement in either group was the use of a funnel. All surgeries were performed by the same surgeon, in the same in-office operating suite, using the same antibiotic irrigation rinse (single antibiotic rinse of clindamycin or cefazolin depending on allergies), skin prep with betadine, and distribution of implant manufacturer.

The occurrence of a unilateral grade III or IV capsular contracture was considered one single event in each group, whereas bilateral capsular contractures postoperatively were considered to be two events. Statistical analysis of the data found was using the Fisher’s exact test. To eliminate the number of confounding variables, patients having any type of breast augmentation surgery after any radiation or chemotherapy were excluded from the study. The periareolar incision type was isolated and studied based on increased historical contracture rates.

The study design presented was approved by an Institutional Review Board at Georgetown University, and the study practices used followed all the guidelines of the Department of Health and Human Services Regulations for the Protection of Human Subjects.

## RESULTS

A total of 237 patients having periareolar breast augmentations from July 1, 2008, through August 31, 2017, were followed. Of those, 20 patients had augmentations before December 10, 2010. N = 20 patients were treated with periareolar augmentation, 13 of which had implants placed submuscular (65%), and 7 (35%) were placed subglandular. There were 217 patients having periareolar breast augmentations using the Keller funnel no-touch technique. Of these 217 patients, 202 (93%) had implants placed subpectoral, and 15 (7%) were placed subglandular.

In group A, those having augmentations without a Keller funnel (n = 20), there was a response/follow-up rate of 75%, leaving 15 patients included in the study results in group A (4 patients responded, but were not able to follow-up and were therefore excluded). Of this group, there were 3 grade III or IV capsular contractures. In group B (n = 217), there was a response/follow-up rate of 70% leaving 151 patients included in the study results for group B (17 patients responded, but were not able to follow-up and were therefore excluded). In group B, there were 4 reported grade III or IV capsular contractures.

Of the 15 patients included in group A for this study, there were 30 total implants placed. Of the 151 patients included in group B, there were 300 implants placed. These results give the incidence of capsular contracture as 10% and 1.3% for groups A and B, respectively (Table 1). There was an 87% reduction in the incidence of capsular contracture, which is statistically significant, at a significance level of 0.05, in showing that the use of a Keller funnel no-touch technique decreases the rate of capsular contracture in patients having periareolar breast augmentations (*P* = 0.0019). By logistic regression, the incidence rate ratio was 4.5 (95% confidence interval, 0.6592–26.5996)

**Table 1.**
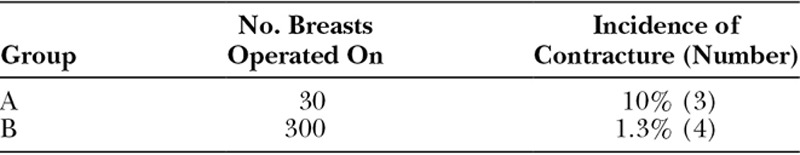
Incidence of Contracture per Breast in Groups Having Periareolar Breast Augmentation

## DISCUSSION

Capsular contracture is 1 of the most common complications after breast augmentation. This study evaluated 237 patients, in an isolated plastic surgery practice, treated with periareolar breast augmentations. Many factors contribute to the occurrence of capsular contracture including incision type, pocket placement, implant type/texture, radiation damage, and so on. Periareolar incision for breast augmentation has been implicated as an incision that leads to significant rates of capsular contracture, ranging from 2.4% to as high as 18.9%.^26,27,32^ Implantation without a funnel through a periareolar incision leads to these higher rates as the implant comes in contact with the skin and milk ducts, which can house bacteria not susceptible to skin prep solutions.^16^ The bacteria coming into contact with an implant is the likely source of the biofilm that begins to form around the implant, ultimately leading to a contracture. It is known and documented that contracture can occur anytime during the lifetime of an implant, but Stutman et al.^17^ reports that a majority of capsular contractures, in periareolar breast augmentation, occur within the first 12 months. The average postoperative follow-up time for patients in this study was 23 months. Patients included in the study were followed for a minimum of 1 year prospective from implantation date and will be continued to be monitored for the lifetime of their implants. In a larger study, it was reported that 36% of contractures that will occur within 10 years, will occur in the first 12 months, and that 65% will occur within the first 4 years.^32,33^ Of the total 166 patients included in this study, 93 patients (56%) were 4 or more years postoperative at the conclusion of data acquisition and follow-up. All the capsular contractures in group A occurred within 4 years of implantation.

In a study done by Spear and Murphy,^32^ 26.3% of implants placed in a subglandular pocket resulted in capsular contracture, whereas only 15.9% of implants placed in a submuscular pocket resulted in capsular contracture. Subglandular implant placement is shown to have increased rates of capsular contracture.^33^ In both groups A and B for this study, more implants were placed in submuscular pockets (65% and 93% for groups A and B, respectively). Only 1 of the capsular contracture in group A resulted from subglandular placement, whereas none of the reported capsular contractures for group B resulted from subglandular implant placement.

Many grade I and II contractures can be addressed through message and anti-inflammatory medication. Anti-mast cell therapy is also a promising area of treatment as mast cell hyperplasia is common within fibrotic tissue and mast cells are known to synthesize many profibrotic mediators.^28^ This study included symptomatic grade III and IV capsular contractures requiring surgical intervention. The overall results of this study indicate that periareolar breast augmentations performed with the assistance of a Keller funnel for implantation have a lower rate of capsular contracture (1.3%) than those without the assistance of a funnel (10%). The group augmented with a funnel had a rate of capsular contracture that was considerably lower than historical rates.^7,15,16,18,20,32^ It is meaningful to note that the 4 patients having contractures resulting from surgeries before the implementation of funnel use, acquired contracture within 2 years postoperatively. Upon reaugmentation, after the implementation of funnel use for insertion, none of the 4 contractures recurred. Additionally, 4 patients in the funnel group were new patients who presented with capsular contracture. Each of these patients had augmentations before the first edition Keller funnel was approved for use in 2009. After having surgery, in which the surgeon used a Keller funnel, none of the patients had any recurrence of contracture. It is also important to note that in 1 incident of contracture, in the group having periareolar breast augmentations with a Keller funnel, the patient was taking Accutane and did not inform the provider. When the patient was taken off Accutane, the breast did soften, although surgical intervention was still required.

The response rates in this study were remarkably high due to physical examination, e-mail, and telephone correspondence being compiled. Typical response rates for e-mail correspondence surveys are reported to be 54.3%, while in this study the rates were between 70% and 75%.^30^

Because of the many controlled variables, including surgeon, patient pool, antibiotic irrigation, implant type, and surgical suite, this lack of recurrence after Keller funnel use is likely due to the no-touch technique afforded by the funnel. Confounding variables were controlled for by ensuring the sole surgical difference between patients placed in group A versus group B was whether or not the patients’ implants were inserted with a Keller funnel. A criticism of single antibiotic irrigation in the surgical technique is valid, yet would have impacted both study groups. Subsequent augmentations have used triple antibiotic irrigation as recommended by Adams et al.^31^

The goal of this study was to evaluate if enhanced no-touch technique with a Keller funnel reduces the rate of capsular contracture in periareolar augmentation. The limitations of this study include the small size of the control group due to early adoption and consistent use of the funnel technique by the surgeon. This study did not separate those having breast augmentations with smooth versus textured implants, as 98% of implants in this study were smooth implants. Although the difference in the rate of capsular contracture between patients in group A (10%) versus group B (1.3%) was statistically significant, this study is limited by the small number of patients who had periareolar breast augmentations before funnel implementation limits this study. However, if there were more patients in this group and the implant sample size was 50, compared with the true implant sample size of 30 in this study, the rate of contracture in this group would be 6.0%, which is still statistically different from the rate of contracture in patients having augmentations after funnel implementation (*P* = 0.0308). The results in the group having augmentation without the use of a Keller funnel were compared with the Allergan Natrelle Round Silicone Core Study, as a majority of the implants placed in this study were Natrelle implants. The rate of capsular contracture was found to be 18.9% after 10 years.^32^ The average follow-up time for group A was 7.2 years, whereass the rate of capsular contracture was 10%. This comparison shows that our rate of capsular contracture for group A was not abnormally high compared with historical rates found in long-term studies.

The statistics in this report were calculated using MedCalc statistical software, MedCalc for Windows, Version 18.2.1 (MedCalc Software, Ostend, Belgium)

## CONCLUSIONS

This study shows that the use of a Keller funnel intraoperatively can statistically significantly reduce the occurrence of capsular contraction in periareolar breast augmentations, which is a major concern in this technique.

A criticism of this study is its retrospective nature, but the resources required to do this as a prospective study, are beyond those available to this research group, with results available a decade from now. As such, this study could be considered a pilot for future larger, prospective study. A strength of this study is that the only variable among surgeon, faculty, and techniques that is being evaluated is the no-touch addition of the funnel. Further studies could study the efficiency of the funnel in preventing capsular contracture in implants placed in alternative pockets through trans-axillary or inframammary fold incisions.
